# C-peptide persistence in type 1 diabetes: ‘not drowning, but waving’?

**DOI:** 10.1186/s12916-019-1415-5

**Published:** 2019-09-27

**Authors:** R. David Leslie, Tanwi Vartak

**Affiliations:** Department of Immunobiology, Blizard Institute, 4 Newark Street, London, E1 2AT UK

**Keywords:** Type 1 diabetes, Stratification, Gene risk scores, Proinsulin, C-peptide, Insulin secretion

## Background

One hundred years ago, diabetes was considered to be one disease, although with marked clinical heterogeneity. Adults who developed diabetes could often manage for years with diet alone, but children usually died within 6 months of diagnosis [1]. Once discovered, insulin was used successfully to treat these children. Thereafter, these children were classified as having insulin-dependent diabetes, which was later re-termed as type 1 diabetes with a dependence on insulin to survive. Patients with all types of diabetes were classified as having non-insulin-dependent or type 2 diabetes, although many eventually required insulin treatment themselves, despite their less aggressive disease course. The development of radioimmunoassays for insulin, proinsulin and its cleaved section C-peptide, only tended to confirm that children with diabetes had little or no insulin, while adults often had substantial amounts. This binary approach to diabetes stratification i.e., that patients do or do not secrete insulin has recently been challenged, notably by exploiting immunogenetics and more sensitive protein assays to define the diabetes spectrum and its heterogeneity [1–3].

## C-peptide spectrum

In their BMC Medicine article, McKeigue and colleagues [4] explored detectable C-peptide in a large cohort from Scotland, representing a cross-sectional analysis of one-third of the population diagnosed with type 1 diabetes over age 16 years. Patients were diagnosed both clinically, by exclusion of other causes, and with one or more diabetes-associated autoantibody. Detectable C-peptide levels varied substantially, with age and disease duration being key variables: C-peptide was lowest in the youngest 15 years from diagnosis (19%) and highest in the oldest, close to diagnosis (92%). Genetic analysis using single nucleotide polymorphisms indicated some heritability for C-peptide variance (26%) [4]. As expected, both human leucocyte antigen (HLA) and insulin gene regions reflected the most aggressive disease process. Persistence of C-peptide was associated with gene risk scores for both type 1 and type 2 diabetes, but also strikingly with HLA regions, not accounted for by high gene-risk HLA serotypes. In other words, the genetics of C-peptide persistence may be distinct from the genetics of C-peptide loss, invoking multiple genetic networks linked to both type 1 and type 2 diabetes.

This paper opens a can of worms regarding our understanding of diabetes and its stratification. The apparent categorical difference between the two major types of diabetes has been challenged, with the realisation that some adult-onset type 1 diabetes cases may not initially need insulin treatment and can have similar C-peptide levels as in type 2 cases [1, 3]. The characteristic type 1 diabetes inflammatory infiltrate, or insulitis, turns out to be not so prevalent as thought, and has been dubbed ‘the elusive lesion’ [5]. Indeed, islet beta-cell function and mass may be compromised in multiple ways, e.g. apoptosis and de-differentiation. There may also be many mechanisms to explain the persistence of C-peptide secretion, including new growth, trans-differentiation with the appearance of islet delta cells or, perhaps, distinct beta-cells, impervious to aggressive autoimmune attack [5]. However, any explanation must account for the remarkable spectrum of C-peptide, in which decline follows a pattern of ‘younger, faster, greater’, while C-peptide persistence is also associated with genes distinct from type 1 diabetes susceptibility genes, implicating type 2 diabetes networks [4].

## ‘Stunned’ beta-cells

At first glance, there appears to be a hierarchy of beta-cell dysfunction. We now know that proinsulin is retained, even as C-peptide response declines. However, when C-peptide is low or absent, both C-peptide and proinsulin fail to respond to stimuli [6]. Given histological evidence that beta-cells persist long after diagnosis, the question arises: are these beta-cells ‘sleeping’ and could they be aroused? In other words, are they ‘stunned’, or functionally dead [5, 7]? The fact that proinsulin levels are detectable, even when C-peptide is not, supports the former argument [6]. Since proinsulin, C-peptide and insulin are secreted in equimolar quantities, but insulin is largely extracted by the liver, increased proinsulin:C-peptide (PL:CP) ratios imply accumulation and secretion of inadequately processed proinsulin, a hallmark of endoplasmic reticulum (ER) dysfunction. A cell that is unable to respond to its primary stimulus is called a ‘stunned’ cell and has the intrinsic capacity to restore competence [7]. In the context of diabetes, one feature of such ‘stunned’ cells, is that there is not only a mismatch between insulin secretion and glucose stimulus, but also that the change can recover. ‘Stunned’ beta-cells are probably a feature of both type 2 and type 1 diabetes and can anticipate and predict both types [7]. Such ‘stunned’ cells are conceptually distinct from ER stress, although both imply a dysfunctional beta-cell. Indeed, evidence of ER stress, using this PL:CP ratio, can be found in children at risk of type 1 diabetes and, in them, predicts the disease, especially in children under the age of 10 years [6, 7].

## Clinical implications

Many years ago, we and others found the same altered PL:CP ratio: before type 1 diabetes, but also in identical co-twins of children with type 1 diabetes and in non-HLA identical siblings, who were themselves estimated to be at low disease risk [8, 9]. Such an altered PL:CP ratio also predicated type 1 diabetes [10]. Despite using different immunoassays, these changes in PL:CP ratio give the results coherence, although none can be certain it was entirely intact proinsulin that was being measured [6, 8–10]. The idea that changes in PL:CP ratio could be familial raises the possibility that shared genetic and/or environmental effects play a role in beta-cell susceptibility to damage. That much would be consistent with the evidence in the present paper that persistence of C-peptide, as against decline, is both genetically determined and associated with type 2 diabetes gene risk [4].

## Conclusions

The present data open new avenues for research and hints at various pathways that might have a differential impact on disease risk, rate of disease progression and failure of the disease process to eliminate all beta-cells in the pancreatic islets. Whether sleeping or dead, the possibility that some cells are ‘not drowning, but waving’, suggests restoration of beta-cell function may yet be feasible.

## Data Availability

Not applicable.
